# Characterizing the regulatory logic of transcriptional control at the DNA sequence level by ensembles of thermodynamic models

**DOI:** 10.1093/bioinformatics/btaf534

**Published:** 2025-09-24

**Authors:** Alan Utsuni Sabino, Drielly de Moraes Guerreiro, Ah-Ram Kim, Alexandre Ferreira Ramos, John Reinitz

**Affiliations:** Departamento de Radiologia e Oncologia, Faculdade de Medicina, Universidade de Sao Paulo, Sao Paulo 01246 903, Brazil; Instituto do Cancer do Estado de Sao Paulo Icesp, Hospital das Clinicas da Faculdade de Medicina da Universidade de Sao Paulo FMUSP HC, Sao Paulo 01246-000, Brazil; Escola de Artes, Ciencias e Humanidades, Universidade de Sao Paulo, Sao Paulo 03828-000, Brazil; School of Life Science, Handong Global University, Gyeong-Buk 37554, Republic of Korea; Departamento de Radiologia e Oncologia, Faculdade de Medicina, Universidade de Sao Paulo, Sao Paulo 01246 903, Brazil; Instituto do Cancer do Estado de Sao Paulo Icesp, Hospital das Clinicas da Faculdade de Medicina da Universidade de Sao Paulo FMUSP HC, Sao Paulo 01246-000, Brazil; Escola de Artes, Ciencias e Humanidades, Universidade de Sao Paulo, Sao Paulo 03828-000, Brazil; Department of Ecology & Evolution, University of Chicago, Chicago IL 60637, United States; Department of Statistics, University of Chicago, Chicago IL 60637, United States; Department of Molecular Genetics and Cell Biology, University of Chicago, Chicago IL 60637, United States

## Abstract

**Motivation:**

Understanding how the genome encodes the regulatory logic of transcription is a main challenge of the post-genomic era, and can be overcome with the aid of customized computational tools.

**Results:**

We report an automated framework for analyzing an ensemble of fits to data of a thermodynamics-based sequence-level model for transcriptional regulation. The fits are clustered accordingly with their intrinsic regulatory logic. A multiscale analysis enables visualization of quantitative features resulting from the deconvolution of the regulatory profile provided by multiple transcription factors interacting with the locus of a gene. Quantitative experimental data on reporters driven by the whole locus of the *even-skipped* gene in the blastoderm of *Drosophila* embryos was used for validating our approach. A few clusters of highly active DNA binding sites within the enhancers collectively modulate *even-skipped* gene transcription. Analysis of variable enhancers’ length shows the importance of bound protein–protein interactions for transcriptional regulation. The interplay between activation and quenching enables function conservation of enhancers despite length variations.

**Availability and implementation:**

The transcription factor level data used for performing the reported study is accessible in the input files in Zenodo and GitHub as well the full code. Additional data from formerly FlyEx database will be available under request.

## 1 Introduction

The interpretation of models of natural phenomena containing large numbers of parameters and multiple scales is a central issue in 21st century science. Although correct predictions by such models are obviously of great utility, their central purpose is to increase human understanding of complex natural phenomena. This issue is particularly acute in systems biology, where a key problem is the elucidation of the control of a variety of processes by DNA sequences, notably including transcription and development. Elegant fundamental studies of developmental processes by parameter free models have been performed (Bauer *et al.* 2021, Bialek 2024, McGough *et al.* 2024, Sokolowski *et al.* 2025), and hypothesized the existence of quantitative limits to be satisfied by the molecular machinery governing the synthesis of gene products in developing *Drosophila* embryos. However, as this approach cannot address the inherently parametric effects of DNA sequence, it does not shed light on how the astonishing optimality observed in biological processes is reached. Indeed, this is also an issue of models exclusively relying on machine learning techniques, which do not address the specific application-domain internal structure of natural science models, and are hard to interpret even in the presence of effective prediction. Therefore, constructing phenomenological models aiming at a description of the processes underlying the regulation of expression in metazoans is a main scientific challenge of the post-genomic era.

Typically, models of complex phenomena with many parameters are constrained by—fit—to observed quantitative data, which itself is subject to experimental uncertainty. Ideally, one aims to obtain a sufficiently large number of fits for the parameters of the model constrained by data. That essentially comprises a posterior distribution, a picture that is true whether or not the fits are obtained by an explicit Bayesian method. In simple problems, standard statistical methods can be used. In more complex situations with many parameters, one is typically interested in what classes of fits are compatible with the data. For that, machine (or statistical) learning techniques may be useful for dimensionality reduction and identification of fits of a given class. When those classes are assessed with the aid of a phenomenological model, they enable building a scientific interpretation about the mechanisms underpinning complex phenomena. Hence, developing automated procedures to improve the identification of fits coherent with the experiments is a key goal of systems biology because its subjects have many parameters and require ensembles of fits of phenomenological models to be understood (Small and Arnosti 2020).

In this article, we present a computational framework for the analysis of a multi-parameter and multiscale phenomenon. It is employed to aid in the interpretation of the genomic regulatory mechanisms inferred from ensembles of fits to expression data obtained from a sequence-level model for transcriptional regulation. This model has been validated by experiments in early *Drosophila* embryos (Janssens *et al.* 2006, Kim *et al.* 2013, Martinez *et al.* 2014, Barr *et al.* 2017, Barr and Reinitz 2017, Masuda *et al.* 2024). It was also used to understand transcription regulation in Murine erythropoiesis (Bertolino *et al.* 2016), and human cells (Kang and Kim 2024). The modulation of promoter activity is non-linearly modeled as a diffusion limited Arrhenius law, with the height of the energy barrier being computed by a multi-layered mathematical structure that represents the multiple couplings among transcription factors (TFs) and DNA regions (Reinitz *et al.* 2003). Pioneer factors are assumed to be homogeneously distributed within the embryo, while hubs of freely diffusing proteins are not considered.

Our framework is validated using cellular resolution quantitative gene expression data from blastoderm of the *Drosophila melanogaster* embryo, at time class 6 (∼36 min after the beginning) of cycle 14 of development, along the anterior–posterior axis (A–P) (Surkova *et al.* 2008a). During this stage, the *Drosophila* embryo is a syncytium, and nuclei along the A–P axis of the embryo have their fate determined with single-cell precision by gene regulatory mechanisms. These spatially distinguishable regulatory profiles enable the use of the early *Drosophila* embryo as a microarray. For instance, maternal and gap genes (so-called because of gaps formed in developing mutants) modulate the expression of the pair-rule genes. The latter produces a seven stripes pattern of expression along the A–P axis, i.e. crucial for the formation of the segments found in the adult fly. The eventual spatial variability of the early concentrations of the transcription factors governing segmentation is canalized towards specific patterns at time class 6. During this stage, *even-skipped* (*eve*), a pair-rule gene, is regulated only by maternal and gap genes, and in the region of stripes 2–7 its expression along the A–P axis is uncoupled from the dorsal–ventral one. That feature simplifies the mathematical properties of the model for transcriptional regulation of *eve’*s basal promoter by its whole locus. Hence, we are capable of fitting experimentally obtained data on *eve* mRNA levels using our model. We expand the analysis initiated in Barr and Reinitz (2017), in which a few model fits, each involving 32 parameters, were analyzed, and inspection was employed for determining the compatibility of genomic regulatory activity with experimental data at an enhancer-level resolution. Similarly, few fits to expression data guided by a sector of the *eve* locus were found with genomic resolution down to individual binding sites (Kim *et al.* 2013). An automated unified framework to provide an ensemble of experimentally compatible fits for the *eve* locus DNA sequence at genomic resolution down to binding sites is missing.

Here, we report a toolset for automated production of ensembles of several thousand fits to data, filtering of fits lying within the uncertainty of expression data, and visualization of regulatory mechanisms producing a given expression pattern down to individual binding sites according to available TFs. Our approach provides a statistics-based procedure for reducing the search region within the parameter space that leads the optimization algorithm to critically increase the proportion of biologically compatible fits to data in an ensemble. Clustering of the fits demonstrates the existence of redundant regulatory logic underpinning an expression pattern. Functional clusters of neighboring binding sites for activating and repressing TFs are shown as drivers of the action of an enhancer. We also show that bound protein–protein interactions are key processes governing promoter activity. In Section 2, we discuss the biology of the sequence-level model for transcriptional regulation, while its mathematical formulation is presented in the Supplementary Information  (SI), available as supplementary data at *Bioinformatics* online. The automated method is described in Section 3. In Section 4, we present the biological insights provided by our framework.

## 2 Materials and Methods

**Figure 1.**
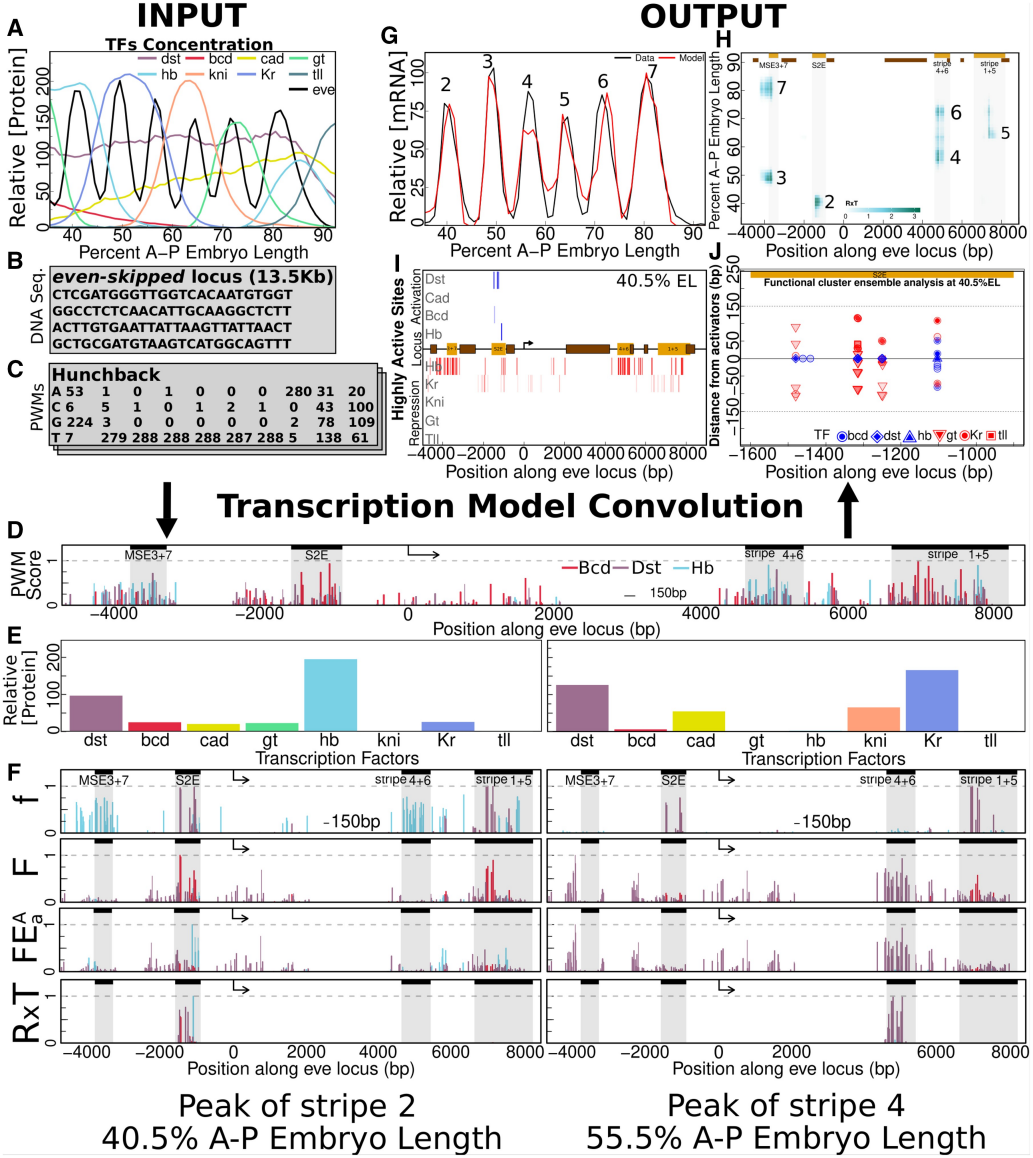
Transcription model convolution scheme. TFs concentration, DNA sequence, and PWMs are the model’s inputs (A–C). Our model has multiple layers that encode the phenomenology of transcription regulation in fruit fly embryos. (D) We represent the first layer with Bcd, Dst, and Hb normalized PWM scores. (E) Protein concentration in two different EL positions, 40.5% and 55.5%, along the A–P axis. (F) Binding sites activities across the layers showing the occupancy (*f*), effects of coactivation (*F*), activation efficiency ($FE_{a}^{A}$), and enhancer competition (R×T) at the EL positions corresponding to the TFs mentioned in (D). (G–J) Multi-resolution outputs obtained from the model in which the predicted *eve* expression pattern (red line) (G), enhancer regions (H), highly active binding sites (I), and functional clusters (J) are shown.

The sequence-level model for transcriptional regulation combines (i) a thermodynamic picture of the binding of TFs to DNA together; (ii) phenomenological rules for the biological function of TFs once bound. The former picture denotes a class of models (Reinitz *et al.* 2003, Janssens *et al.* 2006, Segal *et al.* 2008, Fakhouri et al. 2010, He *et al.* 2010, Kazemian *et al.* 2010, Kim *et al.* 2013, Martinez *et al.* 2014, Samee and Sinha 2014, Bertolino *et al.* 2016, Sayal *et al.* 2016, Barr *et al.* 2017, Barr and Reinitz 2017), while the latter class expresses well-known experimental results in computable equations. Figure 1 summarizes the sequence-level model for transcriptional regulation and the biological implications built upon its outcomes.

Figure 1A–C shows the *inputs* which we use to characterize the regulatory profile of transcription: (1A) nuclear resolution quantitative data on the concentrations of the minimal set of TFs driving *eve* transcription along A–P axis at time class 6, namely, *Drosophila*-STAT (Dst) [as presented in Kim *et al.* (2013)], and Bicoid (Bcd), Caudal (Cad), Hunchback (Hb), Giant (Gt), Kruppel (Kr), Knirps (Kni), Tailless (Tll), along with the spatial pattern of expression of *eve* (Surkova *et al.* 2008a); (1B) the whole sequence of the locus of *eve*, and experimental information on its chromatin accessibility (Li *et al.* 2011, McKay and Lieb 2013); (1C) the position weight matrix (PWM) (Zhao *et al.* 2009) of each TF. The values of the parameters of the model are obtained by application of the simulated annealing (SA) algorithm (Kirkpatrick *et al.* 1983). For the optimization procedure, we label Bcd, Cad, and Dst as activators, repression by Hb, Gt, Kr, Kni, and Tll happens by quenching (Reinitz *et al.* 2003). Though Hb is a quencher, it can effectively behave as an activator if Bcd or Cad is bound to sufficiently close binding sites, a phenomenon called coactivation (Kim *et al.* 2013).

The *processing* of the input is a convolution in which the scores of each binding site along the locus, as estimated from the PWMs of TFs along with their concentrations, govern the activation of the promoter site. Specific DNA regions driving stripe formation along the A–P axis, coincident with experimentally determined enhancers, emerge from this process. We exemplify the role of the first layer of regulation, showing the scores of binding sites for Bcd (red), Dst (purple), and Hb (light blue) along the whole locus (Fig. 1D). This layer of the model does not encode spatial information *per se*, i.e. the scores are the same for all nuclei in the embryo. Positional information depends on the variation of the protein concentrations of the TFs, as exemplified in Fig. 1E, for positions 40.5% and 55.5% of the embryo length (EL) along the A–P axis. Hence, understanding the differential activation of the promoter requires integrating sequence-level information with that of available TFs through hierarchically interacting layers as shown in Fig. 1F. The second layer captures the positional information by means of the occupancy of the binding sites of each TF as shown in the graph for *f*, which, along with the diffusion limited Arrhenius law formulation of promoter activity, contemplates the possibility of TF concentration being limiting. The next three layers phenomenologically quantify the effects of the interactions among bound TFs as described in SI, available as supplementary data at *Bioinformatics* online: coactivation and quenching as encoded by *F* [SI, available as supplementary data at *Bioinformatics* online, Equation (4)]; activation efficiency denoted by $FE_{a}^{A}$ [SI, available as supplementary data at *Bioinformatics* online, Equation (5)]; and enhancer competition indicated by R×T [SI, available as supplementary data at *Bioinformatics* online, Equation (9)]. In each layer, the relative heights of the vertical bars are dependent on position. In a given position, the relative heights of the bars change as we go through the model layers, and that reveals the importance of composition between the binding sites and their bound TFs on driving transcription of *eve*. From here on that composite will be simply referred to as the binding site. Application of the latest layer refines activation regions toward those previously established as enhancers.

Because we employ a sequence-level thermodynamic model, the *output* enables a multi-resolution analysis of the mechanisms underlying *eve* transcriptional regulation. In a previous study, the analysis of the spatial pattern of concentration of *eve* and the respective DNA regions driving transcription was presented (Barr and Reinitz 2017). Figure 1G shows that the transcriptional model prediction (red) reproduces much of the experimentally observed (black) spatial patterns of concentration of *eve* along the A–P axis. Position along the *eve* locus is shown in the horizontal axis of Fig. 1H, while the vertical axis indicates position along A–P. The respective DNA regions activating the *eve* promoter to express stripes 2–7 are highlighted accordingly with their contribution measured as R×T. Here, we introduce an analysis of the binding sites configurations driving expression in a given spatial position. Figure 1I shows the highly active sites contributing to activation of transcription at the peak of stripe 2. The activators are shown in blue and the repressors in red. Those *major activators* of transcription are distinguished by the transparency of the vertical bars, and they concentrate within the Stripe 2 Enhancer (S2E) region. DNA regions beyond S2E are densely occupied by Hb and Kr and prevent, mostly by quenching, the action of activators eventually bound there. A zoom in on the neighborhood of the major activators’ binding sites (blue) at the peak of stripe 2 is shown in Fig. 1J. The vertical axis gives the distance of a neighboring binding site for a quencher or coactivator to the binding site for a major activator. The position along the locus of the site for a major activator is shown on the horizontal axis. Each vertical set of binding sites having a major activating TF at the center along with its quenchers and coactivators between the dashed line forms a functional cluster.

### 2.1 Algorithm

Our goal is to obtain an ensemble of parameter values for the mathematical model shown in the SI, available as supplementary data at *Bioinformatics* online, to generate predictions compatible with experimental observations, which we denote as *biologically compatible fits*. Our framework produces fits to data in two rounds, each involving two thousands applications of the SA algorithm for parameter optimization. Details about the implementation are given in the SI, available as supplementary data at *Bioinformatics* online.

#### 2.1.1 A two-stage procedure for generating an ensemble of fits

Experimental data about the amounts of *eve* transcripts and their respective TFs were used as input for our computational model (Surkova *et al.* 2008a,b, Pisarev et al. 2009). The PWMs for the TFs along with the locus sequence were discussed in Barr and Reinitz (2017). The parameter values for the transcription model presented in the SI, available as supplementary data at *Bioinformatics* online, were obtained by optimization (Fig. S1, available as supplementary data at *Bioinformatics* online) using SA as previously (Jaeger *et al.* 2004, Janssens *et al.* 2006, Manu *et al.* 2009a,b, Kim *et al.* 2013, Barr and Reinitz 2017). The SA algorithm searches for parameter values which minimize the root mean square (RMS) value, $\text{RMS}=\sum_{\nu }{\left[O_{\nu }-M_{\nu }\right]}^{2}$, where $O_{\nu }$ and $M_{\nu }$, respectively, indicate relative *eve* mRNA concentrations as experimentally observed and predicted by the model, and ν denotes the percentage of EL along the A–P axis and runs from 35.5% to 92.5% EL. The SA search region within the parameter space was set based on *a priori* biological information (Barr and Reinitz 2017).

Because the SA is governed by a stochastic process, its output has an intrinsic randomness. Hence, an ensemble of fits to data can be produced to be statistically analyzed with the aim of hypothesizing the regulatory logic underlying a given spatial pattern of gene transcription. Two issues must be approached: (1) Not all fits are biologically compatible, and they must be filtered; (2) Most promising fits may be clustered within the parameter space to determine (2.1) searching regions having a higher probability of generating biologically compatible fits, and (2.2) fits sharing similar regulatory logic. Previously, issue (1) was approached manually and based on the analysis of few good fits to the data. As a consequence, issue (2) has been neglected. Here, we approach both issues by means of an automated procedure.

#### 2.1.2 Two filters enable automated selection of biologically compatible fits

The biological compatibility of the fits obtained by SA is determined by the application of two filters. The first filter selects candidate fits based on the RMS value comparing theoretical and experimentally determined *eve* stripe patterns of transcription. That number enables the selection of fits generating spatial patterns within experimental error.

The fits selected in the previous step will pass through the second filter because the controlling DNA regions being activated may be located beyond those experimentally known (Small *et al.* 1992, Ludwig and Kreitman 1995). Hence, for a given fitting, the program lists the highly active binding sites contributing to transcriptional activity, here set as those causing 80% of the total reduction for transcription initiation along the A–P axis. That percentage is a free parameter of our framework. The biologically compatible fits are those whose transcriptional activity at the peak of stripe 2 had a binding site located within the S2E region among the major activators.

#### 2.1.3 Biologically guided reduction of the volume of the searching region within the parameter space

We performed two rounds of 2000 fits to the data by SA. The first round produced 81 biologically compatible fits. The parameter values of biologically compatible fits are distributed either (a) having most of their values lying within some interval while presenting few outliers; or (b) not having outliers. For the parameters belonging to category (a) we set two reference values $\text{Max}=Q_{3}+1.5\text{IQ}$ and $\text{Min}=Q_{1}-1.5\text{IQ}$, and redefined the limits of search range in terms of Max and/or Min, where $Q_{1}$ ($Q_{3}$), is the first (third) quartile and $IQ=Q_{3}-Q_{1}$. For the parameters belonging to category (b) we used the largest and/or smallest estimated parameter values for defining their search range. The new search ranges were used in the second round of annealing, and we produced 1881 biologically compatible fits, which is a 23-fold change after search region refinement.

#### 2.1.4 Ensemble analysis of biologically compatible models

We seek the most frequent binding sites among the biologically compatible fits to discover the logic of the regulation of transcription of *eve* along the A–P axis as follows. The highly active binding sites driving expression in each position of the A–P axis are collected from each fitting. Then, a weighted sum of the contribution of each activating binding site is performed. The weight of a binding site is its contribution to energy barrier reduction in each fitting, multiplied by the number of times that this binding site appears in all fits. The resulting number is divided by the total number of occurrences of the binding site that appears the most among all biologically compatible fits.

The regulatory logic underpinning stripe formation is proposed after the identification of all binding sites having bound quenchers and coactivators that interact with a specific activator. The combination of each set composed by a highly active binding site, its quenchers, and coactivators, along with their interactive mechanisms, composes what we denote as a functional cluster.

#### 2.1.5 The regulatory logic is further determined based on the clusters of fits

The large ensemble of biologically compatible fits for this data enabled the construction of histograms of parameter values. Those histograms shed light on the multimodality of some parameter value distributions. That prompted us to investigate the formation of clusters of fits among the biologically compatible models. This analysis revealed that the formation of clusters reflects different sets of highly active binding sites driving expression along the A–P axis, and that represents distinguishable regulatory logic.

The analysis of the clusterization of biologically compatible fits was performed using *k*-means. For that, we used a *z*-transformation in each parameter value so that they will all be on the same scale. To define the optimal number of clusters, we vary the number of the *k* parameter in the *k*-means algorithm from 2 to 10 using the package NbClust (Charrad *et al.* 2014) where multiple metrics to assess the number of optimal clusters were employed. A principal component analysis (PCA) was performed using the parameter values of the biologically compatible fits. The first three principal components were used for visualization of the data, i.e. ensembles of parameter values, in a lower dimensional space to enable the inspection of the clusters. Based on the frequency analysis of the best *k* estimated with NbClust and inspection of PCA clusters, the number of clusters to be set in *k*-means was defined as 3.

## 3 Discussion

### 3.1 Automated ensemble analysis shows that model fits recovering enhancer regions at the *eve* locus are in a subdomain of the search space

**Figure 2.**
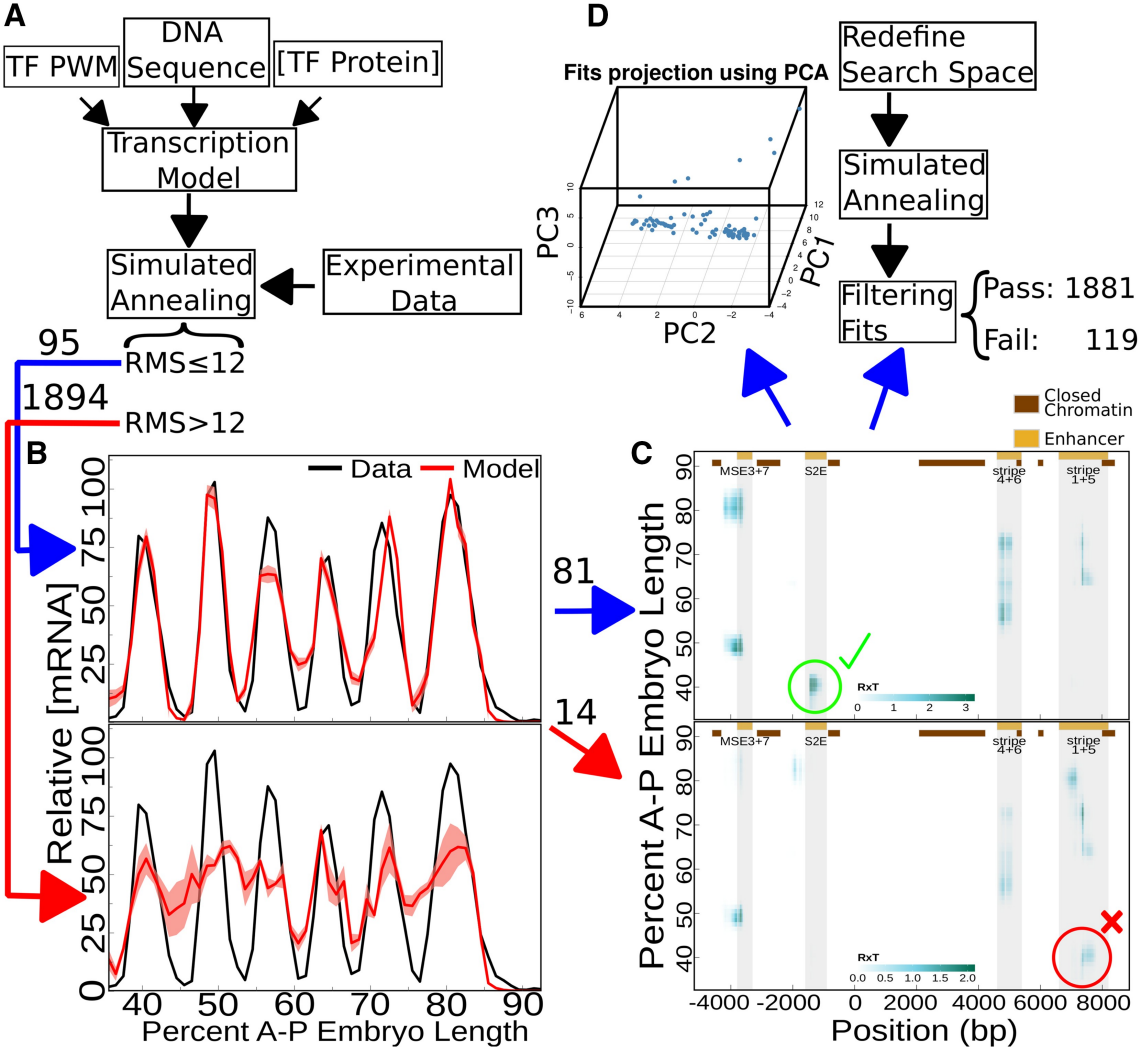
Flow of analysis for obtaining biologically compatible fits. The flowchart presents our computerized method for automated filtering and optimization of fits for the transcriptional model. (A) Simplified scheme of input data and computational process. (B) Mean (red) ± standard deviation (shaded red) of predicted *eve* expression along EL by model fits with low (top) and high (bottom) RMS values—first filter. Experimental data (black) is shown as reference. (C) Prediction of active regions along *eve* locus. The top (bottom) graphs highlight regions of binding sites activation that are compatible (incompatible) with experiments—second filter. (D) 3D Projection of parameter values along the first three principal components obtained after application of PCA to biologically compatible fits (top). Scheme of the process of search space reduction and a new round of SA (bottom).

Figure 2 presents a scheme of a new automated method to generate biologically compatible fits for the sequence-level model for transcriptional regulation and illustrates its usefulness by the analysis of data on transcription driven by the *eve* whole locus. We propose a two-step approach: (i) to generate an initial set of biologically compatible fits, and (ii) to build the distributions of the parameter values to reduce the search space for a second application of our algorithm for finding biologically compatible fits. Inputs are shown in Fig. 2A.

The sequence-level model for transcriptional regulation has 32 free parameters to be optimized by SA fitting the spatial pattern of *eve* stripes (black lines in Fig. 2B). The RMS threshold was set as 12 based on inspection of the graphs (Fig. 2B, top). Only 4.78% (95) of the fits crossed the established threshold used as a first filter. Mean fit predictions (red), with standard deviation, and experimental data (black) are present in Fig. 2B: the top graph shows fits with good expression prediction (RMS ≤ 12), while representative bad predictions (RMS ≥ 20) are shown at the bottom.

*In silico* sequence-level analysis reveals that fits with a good expression pattern can be produced from the regulatory activity of the *eve* locus that do (85.26%) or do not (14.74%), correspond to the experimentally established S2E region (Fig. 2C). The top graph shows regulatory activity from the S2E region, indicated by the shaded region along the DNA sequence positioned between −1600 and −800 basepairs (bp), at the stripe 2 peak of expression, 40.5% EL along the A–P axis (Fig. 2B, top for reference). Stripe 1 + 5 enhancer has regulatory activity at the peak of expression of stripe 2 in the bottom graph, while S2E has no regulatory activity. Hence, it shows a class of fits that are not compatible with the experimental data. After challenging the predicted region regulating the formation of the peak of stripe 2 in comparison with the enhancer region described in the literature, just 4.07% (81) fits produced in the first round of application of SA were selected.

PCA was employed to investigate the biologically compatible fits in a visualizable space having reduced dimensionality, Fig. 2D, top. Inspection revealed the existence of clusters of fits to the data. After applying the reduction in the search space, 94.05% of model fits passed the two quality filters.

### 3.2 Sets of similar fits to data as determined by a clustering algorithm enabled hypotheses about different regulatory logic at the locus

**Figure 3.**
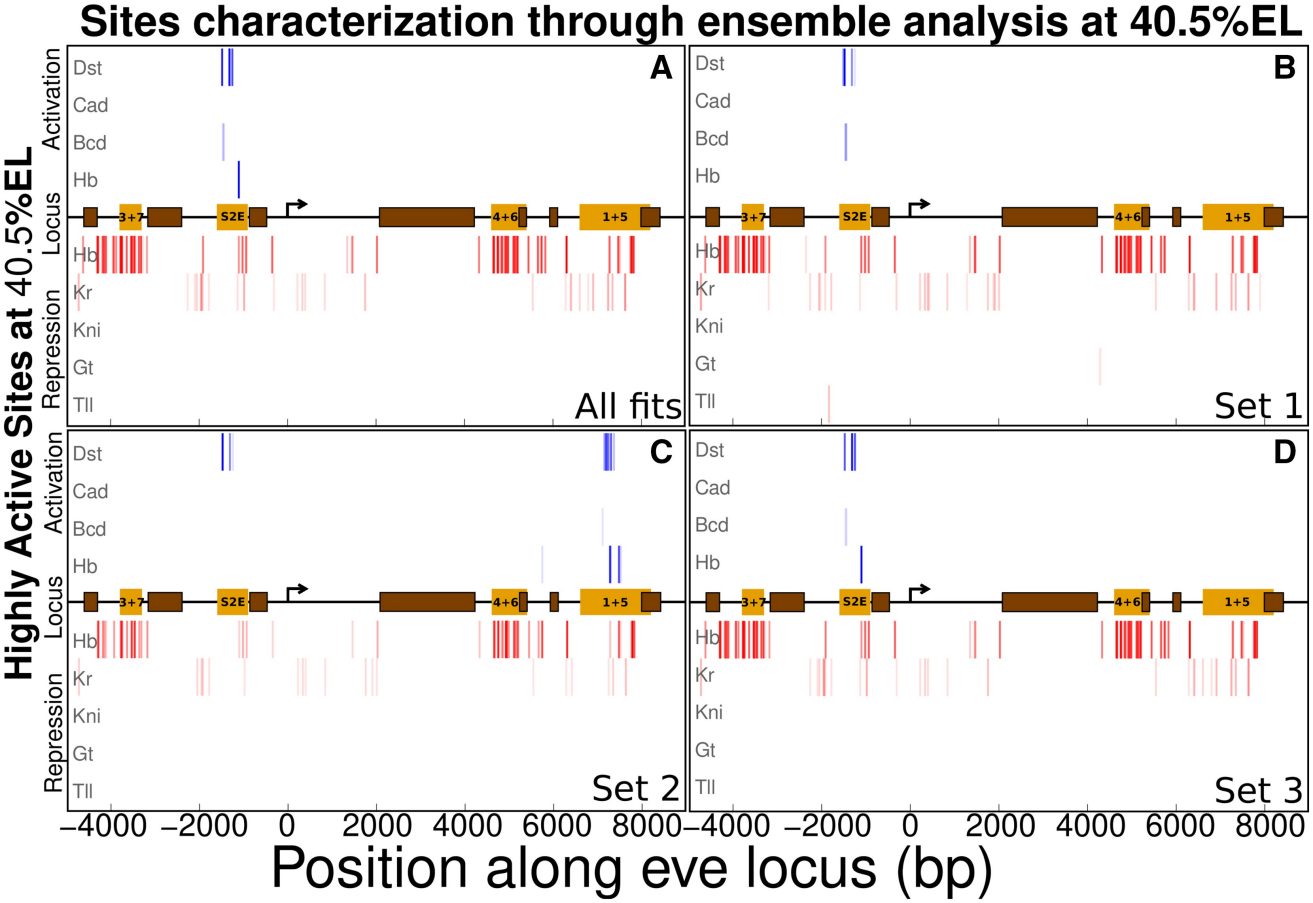
Binding sites interacting to reduce 80% of the energy barrier of transcription activation. The horizontal black line indicates the *eve* locus, with the yellow and brown rectangles, respectively, denoting the enhancers and the closed chromatin regions. Each row along the vertical axis denotes a TF. The activators are indicated by blue vertical bars, while the red bars show the sites for repressors. The more solid the blue (red) bar, the higher is its activation (or repression efficiency, $E_{a_{k}}^{Q}f_{k}^{Q}$). (A–D) The ensemble of fits (A) and its respective sets resulting from clustering parameter values of biologically compatible fits (B–D).

In Fig. 3, we show the highly activated binding sites along the *eve* locus at the peak of stripe 2. Graph A shows the binding site among the highly active ones in the ensemble of fits, while graphs B–D indicate the results of applying our algorithm within the sets of similar fits to data. Our model indicates that Hb, Dst, and Bcd, in decreasing order of contribution, are the main activators of transcription at the peak of stripe 2. Hb is coactivated by Bcd (Kim *et al.* 2013) (graphs A, D). In the ensemble (graph A), set 1 (graph B), and set 3 (graph D) of fits, the highly active sites for activators (blue bars) are concentrated within the region of S2E, while some activation beyond this enhancer is shown in set 2 (graph C). The coincidence of highly active binding sites in graphs A and D is because set 3 has the largest number of fits. Though there are binding sites for activators along the whole locus, they are not active mainly because of quenching. Hb is the major repressor responsible for quenching transcriptional activity induced by non-S2E enhancers. Kr is also important because of its higher affinity to binding sites within the region of the enhancers 1+5 and S2E.

In Fig. 3, the second set of fits has an activation of transcription being induced by the binding sites within the enhancer 1+5. The top contributors for that activation are Dst and Hb, the latter being coactivated by Bcd (see SI, available as supplementary data at *Bioinformatics* online). This set of fits would be discarded for further analysis of the logic of transcriptional regulation of *eve*. But that would be an underestimation of the role of the sets of potentially useful fits if one is willing to employ our methodology in the study of a new system. The clusterization enables one to collect the fits by their similarity. That can be useful in experiments that investigate the logic underpinning the modulation of gene expression by multiple TFs.

Previous experiments have established that the formation of *eve* stripes is driven by enhancers being about ∼700 bp long (Stanojevic *et al.* 1991, Small *et al.* 1992). The results shown in Fig. 3 with a larger window of 1000 bp, however, indicate that only a few activating binding sites are the major contributors to *eve* transcription at the peak of stripe 2, for example. Next, we identify functional clusters encompassing collections of binding sites for repressors, activators, and coactivators whose combined effect underlies the modulation of gene transcription by a controlling DNA region.

### 3.3 Functional cluster characterization aids in understanding the distinctive logic of transcriptional regulation of the locus

**Figure 4.**
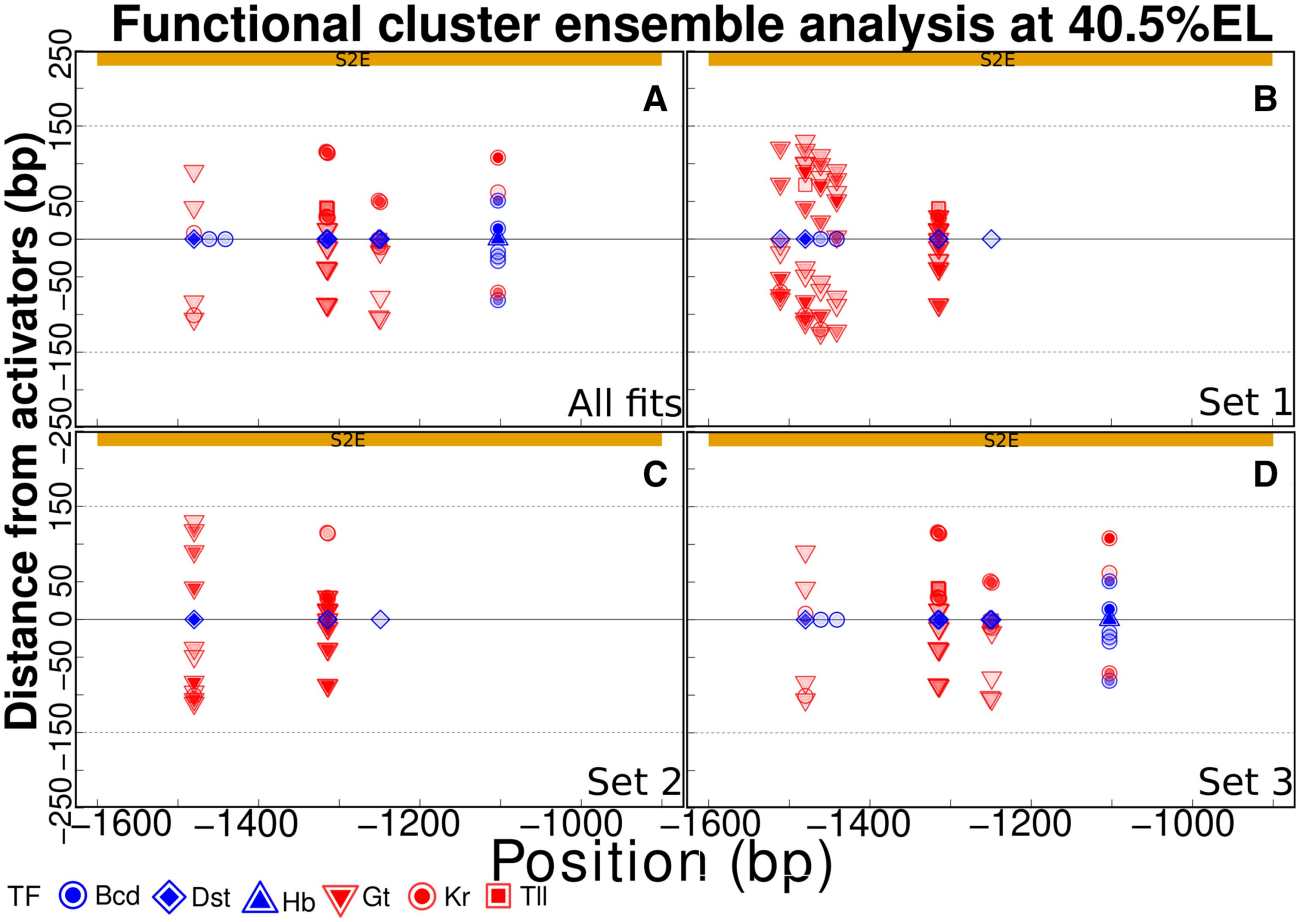
Functional clusters characterization of stripe 2. The top yellow rectangle indicates the region of S2E, and the horizontal axis gives the position along the *eve* locus. The vertical axis indicates the distance, in basepairs, of a binding site to its interacting activator site. The more solid the inner symbol, the stronger the effect of activation (blue) or repression (red). (A–D) Fits ensemble and its sets clustered by parameters.

In Fig. 4, we show the functional clusters that are responsible for an 80% reduction in the energy barrier for transcriptional activation at the peak of stripe 2. Graph A shows the most frequent binding site among the highly active ones in the ensemble of fits, while graphs B–D indicate the outcomes of the application of our algorithm to sets 1–3 of fits to data. In each graph, the functional clusters are labeled according to their energy barrier reduction ranking. The activator in a functional cluster is set as the referential blue symbol in the black horizontal line in the middle of the graphs. In general, the functional clusters are composed of a single activator surrounded by its respective quenchers. However, since Hb is coactivated by Bcd its corresponding functional clusters in graphs 1A and 1D are composed of quenching and activating binding sites. Dst and Bcd binding sites are only surrounded by quenchers, and because we show the stronger repressors, some of their sites are alone. Again, since set 3 of fits has a larger number of components, it has a stronger influence on determining the functional clusters as obtained from all fits (see graphs A and D). In both sets 1 and 2, Hb functional clusters do not appear as the highly active sites within the region of S2E. Note that some Hb functional clusters were highly active in the region of the enhancer 1+5 (see Fig. 3).

The functional cluster analysis of Fig. 4 provides an increased genomic resolution for understanding the regulatory logic as predicted by the sequence-level model for transcriptional regulation in *Drosophila*. It provides a strategy for the selection of the most promising fits to data. For example, graphs A and D show that the coactivation of Hb is a major effect underlying the expression of *eve* at the peak of stripe 2, as previously discussed (Kim *et al.* 2013). Applying our framework to a new system for investigating the main binding sites driving some gene expression pattern might use this class of graphs for identifying the most promising DNA regions to be genetically modified. Graphs B and C show that the spatial patterns of expression of *eve* may be achieved by alternative regulatory logic. This exemplifies the redundancy of the gene regulatory code and challenges experimentalists to find whether these are model artifacts or observable phenomena.

The functional clusters in Fig. 4 may encourage one to investigate whether enhancers of about 700 bp are the outcome of the genomic low resolution of experimental techniques. Previous experiments have shown that *eve* stripe two is driven by the S2E region (Ludwig and Kreitman 1995, Ludwig *et al.* 1998). The functional clusters are spread over the S2E region, though they may not fulfill it completely. Indeed, experimental results show that a smaller region called minimal stripe enhancer 2 may drive *eve* stripe 2 (Small *et al.* 1992), though at lower mRNA levels (Kim 2012). In the SI, available as supplementary data at *Bioinformatics* online, we discussed the window size parameter α which indicates the length of DNA segments competing for reducing the energy barrier of promoter activation. Hence, one may vary α to investigate the capacity of a given DNA segment to induce transcription. Our framework provides the tools for systematically performing such an analysis.

### 3.4 Variation of the length of DNA that influences the promoter shows the importance of bound protein−protein interaction for transcriptional regulation

**Figure 5.**
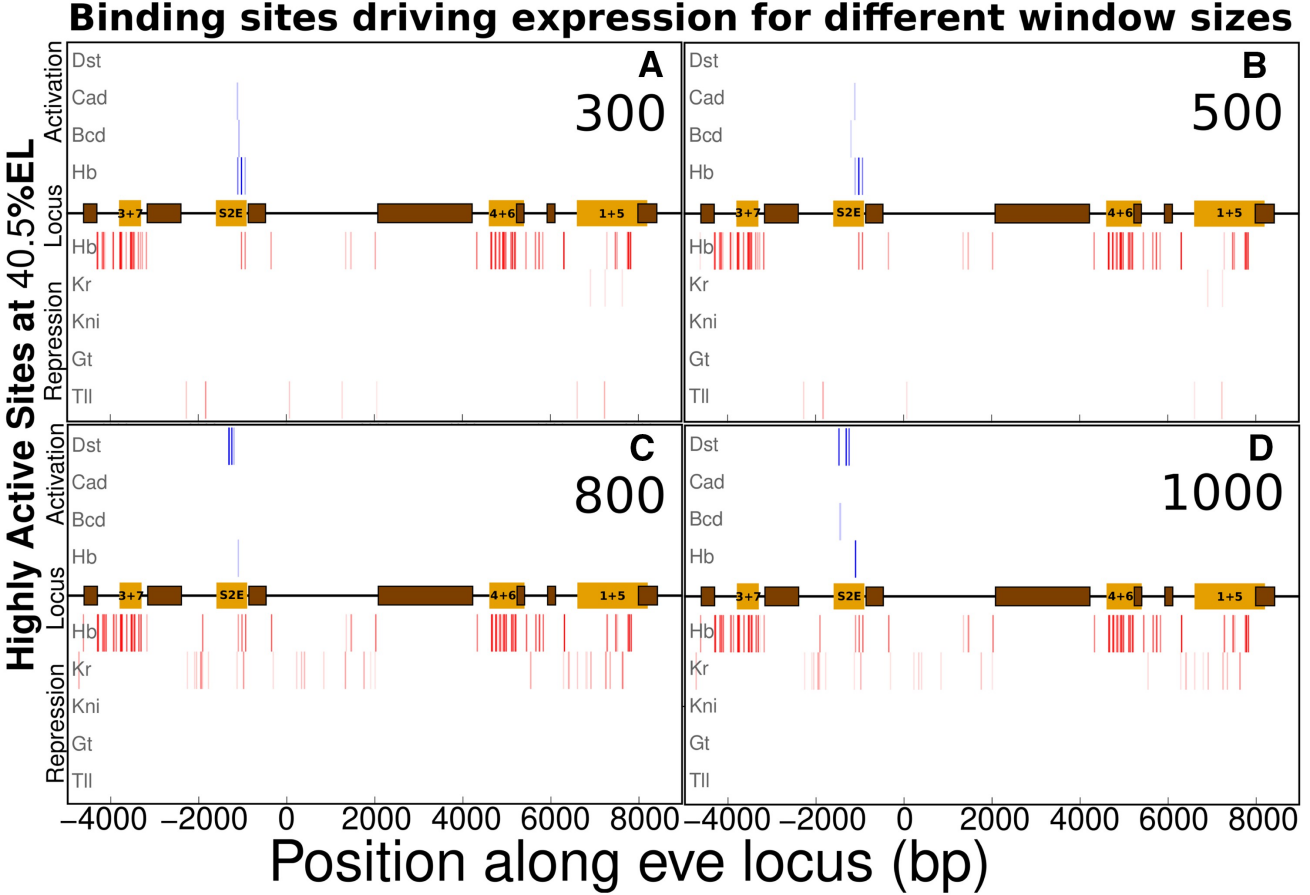
Highly active sites of ensemble fits varying the window size. Binding sites most significant to expression at the peak of stripe 2 are shown for window size parameter with values of 300, 500, 800, and 1000 (A–D).

In Fig. 5, we show the effect of changing the parameter α. Our model fits the *eve* expression while estimating parameter values for each α. That leads to distributions of parameter values being dependent on the window size. As α increases, more binding sites for activators will be available and, hence, more quenching will be needed to keep the transcriptional activity invariant. Shorter interacting regions (300 and 500 bp, Fig. 5A and B) reveal the major importance of Hb, and hence coactivation, to the formation of the peak of stripe 2. Besides, most of the quenching is performed by Hb with a secondary role being played by Tll. As the window size gets longer (800 bp and 1 kb, Fig. 5C and D), Dst binding sites start giving a larger contribution to reduce the activation energy barrier, while coactivated Hb action loses importance. Besides, quenching by Hb and Kr from near the 5′ border of S2E starts to participate in the functional clusters determining expression at the peak of stripe 2. This is related to the emergence of Dst as a major activator along with its quenchers. Quenching from Hb binding sites is mostly conserved as we increase the window size, while some Kr quenching starts to appear as a compensation for the increased DNA region size inducing promoter activity. This behavior of the model unveils the importance of bound protein-to-protein interactions for transcriptional regulation. It reveals the importance of coactivation when we use smaller window sizes, and how the almost uniform concentration of Dst along the A–P axis raises the necessity of additional quenching when α increases. It also shows the limitations of only considering the PWMs as a major guide for establishing the regulation of gene expression in metazoans.

### 3.5 Implications and limitations

Here, we consider the *eve* locus chromatin accessibility as a constant along A–P axis during nuclear cycle 14 (Hannon *et al.* 2017, Cusanovich *et al.* 2018, Haines and Eisen 2018), because we aim to mathematically express experimentally observed phenomena in the simplest possible model for understanding transcriptional regulation. Indeed, only Bicoid cooperativity is considered (Ma *et al.* 1996, Burz *et al.* 1998), though additional interactions, such as cooperativity among other TFs, can be easily hypothesized (or included based on data) within the scope of our framework. The model has an intrinsic non-linear character as it is an approximation of a fully interpretable deep neural network (Liu *et al.* 2020). Interactions or effects such as saturation of DNA sites can be incorporated, respectively, as new layers according to their phenomenology or investigated using a tool available in our framework. Effects of pioneer factors or freely diffusing hubs of proteins are expected to be included as new layers (Nien *et al.* 2011, Mir *et al.* 2017, 2018, Dufourt *et al.* 2018, Yamada *et al.* 2019, Zhang *et al.* 2025). Note that our model reveals the formation of functional clusters of binding sites as an emergent property of enhancers being regulated by several TFs interacting through multiple phenomenological rules. That compensates for the limitations of the PWMs, which predict binding sites as independent entities.

Our model assumes that pioneer factors act homogeneously along the A–P axis. Recent data on *eve* enhancers 2 and 3+7 reinforce the presumption of an ubiquitous action of pioneer factors, though the differential effect of Zelda on the affinity of activating and repressing TFs (Bishop *et al.* 2023, Fig. 2) requires further analysis. In Harden *et al.* (2023, Fig. 1), it is shown that the addition of two Zelda binding sites to a modified enhancer 2 leads to earlier repression of activity of the *eve* promoter, with the fraction of active nuclei reaching about half of its maximum in ∼25 min. This time frame coincides with Gt and Kr reaching high levels of expression (Surkova *et al.* 2008a, Fig. 2), and the faster decay may be the result of increased quenching in enhancer 2. Because our model has already been used to interpret non-anticipated experimental data (Masuda *et al.* 2024), we presume it can be useful again.

Our model was constructed for understanding transcriptional activation (Reinitz *et al.* 2003) and that led us to seek activating binding sites within S2E as a second filter while avoiding being too stringent. Hence, we did not search for sites not bound or binding sites in other enhancers as those require data-driven adjustments in the model. Furthermore, the S2E constitutes a complex model system whose comprehensive experimental characterization (Goto *et al.* 1989, Small *et al.* 1991, 1992, Stanojevic *et al.* 1991, Ludwig and Kreitman 1995, Bothma *et al.* 2014, Bishop *et al.* 2023, Harden *et al.* 2023) prompts it for validating theories on transcriptional regulation (Janssens *et al.* 2006, Kim *et al.* 2013, Prata *et al.* 2016). The application of our framework to a different system will require the adaptation of the filters to properly incorporate experimental pre-established knowledge.

For example, if information about the DNA regions responsible for the regulation of transcription levels is missing, one might use only the result of expression levels (first filter). Then, the possibility of shadow enhancers (Zeitlinger *et al.* 2007, Hong *et al.* 2008, Levine, 2010) would be considered. In a former study, the action of enhancer 2 on the formation of stripe 7 was predicted and experimentally validated (Janssens *et al.* 2006) in agreement with independent studies (Goto *et al.* 1989, Harding *et al.* 1989, Small *et al.* 1996, Staller *et al.* 2015). Here, this possibility is shown in Fig. 2C, and thus, it is fair to hypothesize a shadow enhancer 1+5 participating in stripe 2 production. Chromatin openness within enhancer 1 along the anterior region was shown recently, but the employed method lacks spatial resolution for determining whether it is contributing to the formation of stripe 2 (Haines and Eisen 2018). An experiment should be designed to verify this possibility.

The framework presented here provides one further step toward the construction of a fully automated system for the discovery of gene regulatory regions (Reinitz *et al.* 2003, Janssens *et al.* 2006, Kim *et al.* 2013, Barr and Reinitz 2017). It has been validated in terms of experimental data on *eve*. Validating the techniques here introduced for use in different genes or organisms and incorporating tools for determining chromatin accessibility, quantitative expression data, PWMs, and functional information on TFs, expression data on regulated genes, among others remains a major challenge.

## Supplementary Material

btaf534_Supplementary_Data

## Data Availability

TF level data used for performing the reported study is accessible in the xml file in Zenodo and GitHub. Additional data from formerly FlyEx database will be available under request.
